# Effect of elevated magnesium sulfate on two riparian tree species potentially impacted by mine site contamination

**DOI:** 10.1038/s41598-020-59390-9

**Published:** 2020-02-19

**Authors:** Caroline A. Canham, Ornela Y. Cavalieri, Samantha A. Setterfield, Fiona L. Freestone, Lindsay B. Hutley

**Affiliations:** 1 The University of Western Australia, 35 Stirling Hwy, Crawley, Western Australia 6009 Australia; 2 Charles Darwin University, Ellengowan Drive, Casuarina, Northern Territory 0810 Australia

**Keywords:** Plant physiology, Plant stress responses

## Abstract

Globally, mining activities have been responsible for the contamination of soils, surface water and groundwater. Following mine closure, a key issue is the management of leachate from waste rock accumulated during the lifetime of the mine. At Ranger Uranium Mine in northern Australia, magnesium sulfate (MgSO_4_) leaching from waste rock has been identified as a potentially significant surface and groundwater contaminant which may have adverse affects on catchment biota. The primary objective of this study was to determine the effect of elevated levels of MgSO_4_ on two riparian trees; *Melaleuca viridiflora* and *Alphitonia excelsa*. We found that tolerance to MgSO_4_ was species-specific. *M. viridiflora* was tolerant to high concentrations of MgSO_4_ (15,300 mg l^-1^), with foliar concentrations of ions suggesting plants regulate uptake. In contrast, *A. excelsa* was sensitive to elevated concentrations of MgSO_4_ (960 mg l^-1^), exhibiting reduced plant vigour and growth. This information improves our understanding of the toxicity of MgSO_4_ as a mine contaminant and highlights the need for rehabililitation planning to mitigate impacts on some tree species of this region.

## Introduction

Mining activities have significantly impacted terrestrial and aquatic ecosystems at both local and regional scales^1–3^. Critical to minimising these impacts is appropriate management of waste rock or spoil, that is, the vast quantities of extracted material remaining following segregation and removal of the relatively small amount of the desired substance^4^. Oxidation and weathering of exposed waste rock material can result in acidic and/or sodic leachates^5,6^. Many elements can increase in concentration in groundwater passing through the rock and potentially contaminate the receiving environment. For example, chlorite schists can result in leachate with elevated levels of sulfate (SO_4_), bicarbonate (HCO_3_), calcium (Ca) and magnesium (Mg)^5,7,8^. Long-term site rehabilitation requires knowledge of the tolerance of the receiving environment to run-off contaminants and appropriate management to minimize potential ecological impacts^9^.

Waterways have been impacted from off-mine pollution^9–11^. For example, the U.S. Environmental Protection Agency (USEPA) stated in 2000 that 40% of headwater streams in the western USA were polluted from mining^12^. These streams had significant impacts resulting from the influence of surface or groundwater that flowed through and chemically interacted with rock waste piles^13^, affecting both instream communities and adjacent riparian habitats^14–17^. There has been increasing focus on the prevention, management and mitigation of the potential impacts of mining on rivers and their riparian ecosystem^18^. A healthy riparian zone is critical at both site- and regional-scales, influencing the hydrology and morphology of fluvial systems, supporting terrestrial riparian biota and influencing instream biota^19^.

Mine site rehabilitation and mine abandonment have emerged as major issues in Australia^20,21^ with many current and legacy mines raising questions about long-term site management, particularly of waste-rock and its pollutants^22^. In Australia, provision for mine site rehabilitation is now a requirement of all active mining operations. Ranger Uranium Mine (RUM) occurs in a 79 km^2^ leasehold area surrounded by the World Heritage-listed Kakadu National Park in Australia’s Northern Territory (NT). Uranium mining ceased at RUM in 2012, with all decommission works to be completed by 2026^23^. Rehabilitation is underway with waste rock used as capping for the final landform. Due to the composition of the waste rock, the landform will generate significant magnesium sulfate (MgSO_4_) loads to surface runoff and shallow groundwater^8,23,24^. Elevated concentrations of Mg up to 417 mg l^−1^ and SO_4_ up to 1,770 mg l^−1^ have previously been recorded at a bore near the tailings facility^25^. Furthermore, elevated Mg concentrations (350 mg l^−1^) have been recorded for seepage water expressing in to a tributary (Gulungul Creek) downstream of the tailings facility^26^. These Mg and SO_4_ concentrations are elevated compared to the naturally low background levels in shallow groundwater for the area (9.4–19.2 mg l^−1^ for Mg)^27^. Levels are also low in surface water, for example, at Magela Creek, an ecologically significant water course^26^ which runs through RUM leasehold, Mg concentrations are approximately 0.8 mg l^−1^ and SO_4_ concentrations are approximately 0.4 mg l^−1^, recorded upstream of the mine^28^. Riparian vegetation and aquatic biota of Magela Creek adjacent to the rehabilitated mine site may be at risk of elevated concentrations of MgSO_4_^23,29^.

The potential effect of MgSO_4_ on riparian plants has been identified as a key knowledge need for rehabilitation planning at RUM^30^. Although Mg and S are important macronutrients for plant development^31,32^, elevated levels can have a detrimental impact on plant growth. For example Mg above ≥8.5 mM (207 mg l^−1^) in soil solution was found to impact development of *Arabidopsis thaliana* plants^33^ and sulfate concentrations of 400 mg l^−1^ had a negative impact on an aquatic moss in soft water^34^. The concentration at which plants are impacted differs between species^35^ and varies with site-specific factors, such as the ratio of Ca to Mg in the soil^36–38^. There is significant literature describing physiological effects of Mg deficiencies on photosynthesis and plant growth, but far fewer studies on effects of elevated Mg. Sulfate is generally found to be non-toxic to plants, although at very high concentrations the increased salinity can induce plant osmotic stress^39–41^. There are no studies examining the impacts of MgSO_4_ on native Australian tree species. This paucity of research means there is limited information to guide long-term management of the riparian vegetation of the Magela Creek catchment post RUM closure, or other areas potentially impacted by elevated MgSO_4_ concentrations.

This study assessed the effect of elevated concentrations of MgSO_4_ on two riparian tree species; *Melaleuca viridiflora* Sol. Ex Gaertn. and *Alphitonia excelsa* (Fenzl) Benth. These species occupy different riparian zone habitats within the Magela Creek catchment and both are common downstream from the RUM. The aim was to determine the range of MgSO_4_ concentrations in soil solution where changes to plant physiology and growth could be detected, and to see if responses differed between the two species. To address this aim we undertook three glasshouse trials. Trial 1 assessed the effect of MgSO_4_ concentrations on *M. viridiflora* using a range of concentrations informed by current background MgSO_4_ concentrations in Magela Creek and nearby waterways. Based on the outcome of trial 1, trial 2 assessed the effect of higher concentrations of MgSO_4_ on *M. viridiflora*. Trial 3 used a subset of MgSO_4_ concentrations from the first two trials to determine the effect of MgSO_4_ on the second species, *A. excelsa*.

## Results

There were marked differences in the response to elevated MgSO_4_ concentrations between the two study species. There was no relationship between MgSO_4_ concentration and plant dry mass for *M. viridiflora* in both trial 1 (ANOVA, *F*_2,15_ = 0.04, *P* = 0.96;) and trial 2 (ANOVA, *F*_2,15_ = 0.50; Table 1). By contrast, there was a significant decrease in plant mass with increased MgSO_4_ concentration for *A. excelsa* (ANOVA with Tukey HSD post hoc test, *F*_3,16_ = 9.54, *P* < 0.001; Table 1). At the end of the experiment, mean plant mass of *A. excelsa* individuals in the lowest treatment (5 mg l^−1^) was more than double those in the highest treatment (9,100 mg l^−1^) (56.0 g c.f. 22.3 g, respectively). Plant biomass values were supported by visual assessments of plants throughout the experiment. At the highest treatment concentration (9,100 mg l^−1^), *A. excelsa* had dropped or desiccated leaves by week 10 (Supplementary Fig. 1h), with some leaf loss and desiccation evident in the next highest treatment (3,900 mg l^−1^). (Supplementary Fig. 1g).

**Table 1:** Mean total plant dry mass (1 standard error in parenthesis) across a range of MgSO_4_ treatment concentrations for trials 1 and 2 on *M. viridiflora* (*n* = 6) and trial 3 on *A. excelsa* (*n* = 5).

Trial	Species	MgSO_4_ (mg l^−1^)	Plant dry mass (g)
1MV	*Melaleuca viridiflora*	5	75.7 (6.12)^ns^
		15	80.9 (5.72)^ns^
		960	79.2 (4.88)^ns^
2MV	*Melaleuca viridiflora*	6,000	93.2 (9.85)^ns^
		9,100	98.7 (5.66)^ns^
		15,300	88.5 (5.19)^ns^
3MV	*Alphitonia excelsa*	5	56.0 (6.90)^a^
		960	40.1 (5.57)^ab^
		3,900	27.7 (2.48)^b^
		9,100	22.3 (3.02)^b^

Different letters indicate significant differences between treatments within each trial (1-way ANOVA, *P* = 0.05). Key: 1MV = trial 1 *Melaleuca viridiflora*, 2MV = trial 2 *M. viridiflora*, 3AE = trial 3 *Alphitonia excelsa. P* values; ^ns^*P* > 0.05.

**Figure 1:**
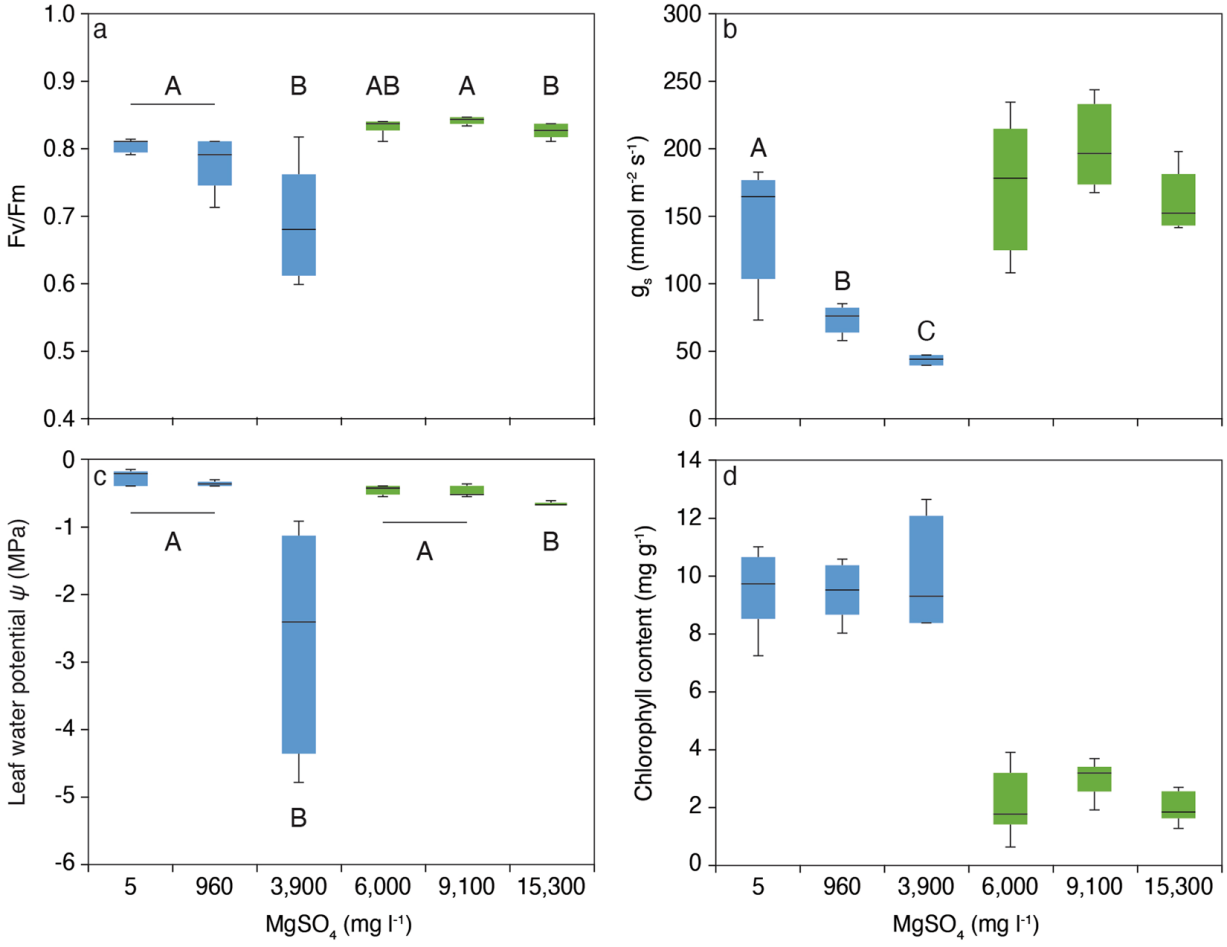
Box-and-whisker plots of leaf-scale physiological measurements for trial 3 *Alphitonia excelsa* (blue; AE) and trial 2 *Melaleuca viridiflora* (green; MV) under different MgSO_4_ concentrations. (**a**) chlorophyll fluorescence (Fv/Fm; *n* = 5 for AE and *n* = 6 for MV); (**b**) stomatal conductance (g_s_; *n* = 4 for AE and *n* = 5 for MV); (**c**) pre-dawn plant leaf water potential (Ψ; *n* = 4 for AE and *n* = 6 for MV); and (**d**) chlorophyll content (*n* = 4 for AE and *n* = 6 for MV). Different capital letters indicate significant differences between MgSO_4_ treatments within each trial (1-way ANOVA, Tukey HSD post hoc test, *P* = 0.05).

Differences in mean plant mass at week 10 were reflected in chlorophyll fluorescence and pre-dawn water potentials. For *A. excelsa*, stomatal conductance decreased with increasing MgSO_4_ concentration, declining from 144.6 m^−2^ s^−1^ in the 5 mg l^−1^ treatment to 42.9 m^−2^ s^−1^ in the 3,900 mg l^−1^ treatment (ANOVA with Tukey HSD post hoc test, *F*_2,11_ = 16.46, *P* < 0.001). Only one *A. excelsa* individual in the 9,100 mg l^−1^ treatment had leaves remaining by week 10 so this treatment was not included in the analysis. For *M. viridiflora* there was little variation in chlorophyll fluorescence, with values ranging from 0.82 to 0.84 Fv/Fm (ANOVA with Tukey HSD post hoc test, *F*_2,15_ = 4.38, *P* = 0.03; Fig. 1a) and there were no significant differences between treatments for stomatal conductance (Fig. 1b). There were no significant differences in chlorophyll content between MgSO_4_ treatments for either species (*A. excelsa F*_2,11_ = 0.08, *P* = 0.923; *M. viridflora* ANOVA *F*_2,15_ = 2.98, *P* = 0.08; Fig. 1d). Overall, mean leaf chlorophyll content across treatments was higher in *A. excelsa*, with an average of 9.6 mg g^−1^ compared with 2.4 mg g^−1^ for *M. viridiflora* in both trial 1 and 2.

For *A. excelsa* predawn water potential was significantly lower at a treatment concentration of 3,900 mg l^−1^ (ANOVA with Tukey HSD post hoc test, *F*_2,11_ = 29.04, *P* < 0.001). At lower concentrations of 5 mg l^−1^ and 960 mg MgSO_4_ l^−1^, *A. excelsa* seedlings did not indicate water stress, however, at 3,900 mg l^−1^ the majority of replicate plants had predawn shoot water potentials lower than wilting point (−1.5 MPa). There was only one replicate in the 9,100 mg l^−1^ treatment due to leaf-loss by the majority of the plants, and again this value was below wilting point (excluded from analysis). For *M. viridiflora* plant water potential was lowest at the highest MgSO_4_ treatment concentration of 15,300 mg l^−1^ (ANOVA with Tukey HSD post hoc test, *F*_2,15_ = 19.97; *P* < 0.001), although values remained above −0.8 MPa, indicating that plants were not water stressed (Fig. 1c).

**Figure 2:**
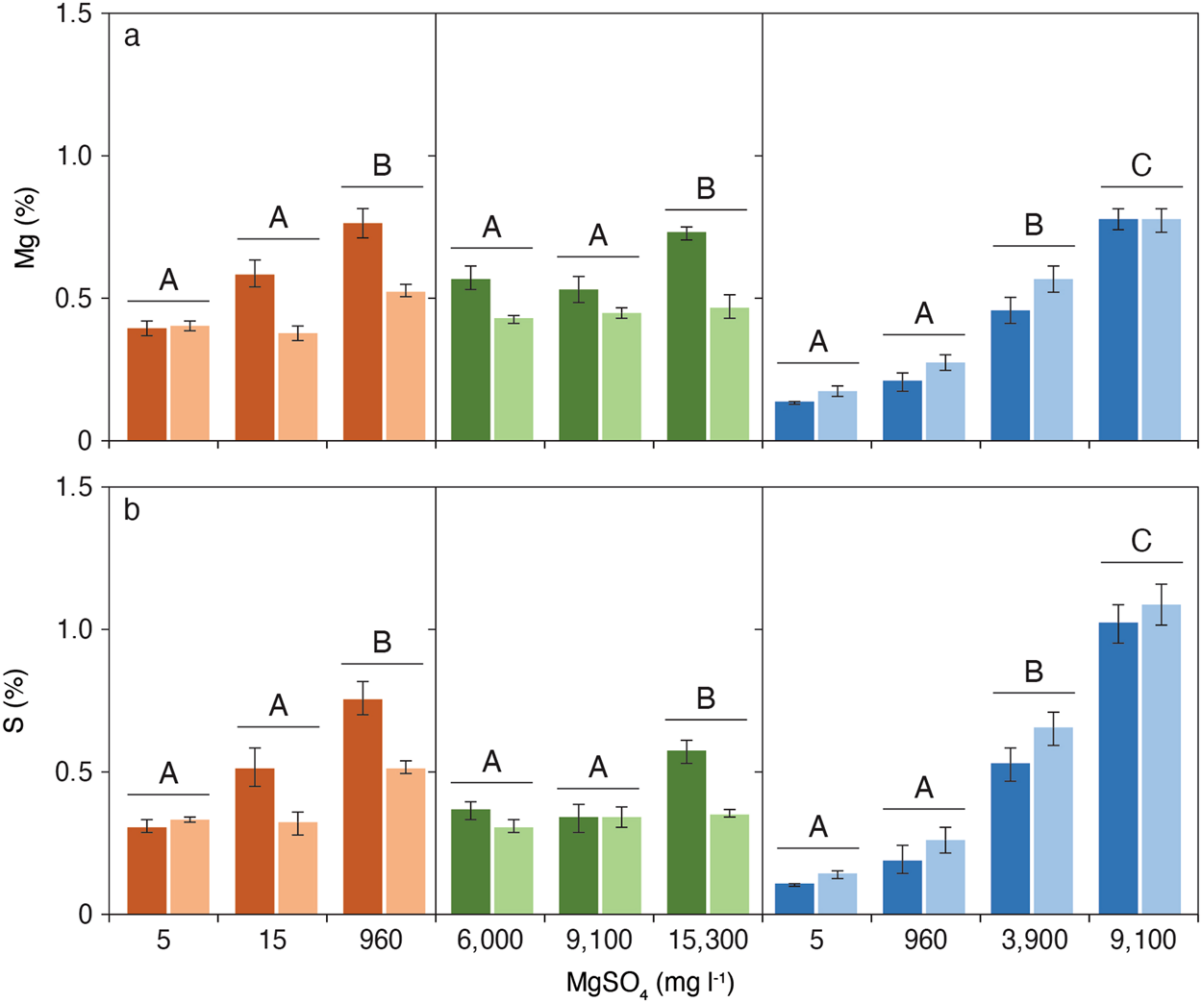
Concentrations of (**a**) Mg and (**b**) S in upper and lower leaves of *M. viridiflora* trial 1 (orange; *n* = 6 for upper and *n* = 5 for lower), *M. viridiflora* trial 2 (green; *n* = 6) and *A. excelsa* (blue; *n* = 5) plants treated with different MgSO_4_ concentrations (mean per treatment with SE). Dark and light colours refer to lower and upper leaves respectively. Different capital letters indicate significant differences between MgSO_4_ treatments within each trial. Upper and lower leaves within each trial were significantly different (2-way ANOVA, Tukey HSD post hoc test, *P* = 0.05).

In each trial there was a general trend of higher foliar concentrations of Mg and S in plants receiving higher concentrations of MgSO_4_ (Fig. 2 and Table 2); however, there were differences in uptake between the two species. For *A. excelsa*, increasing concentrations of MgSO_4_ resulted in a direct increase of Mg and S concentrations in leaves (2-way ANOVA with Tukey HSD post hoc test, *F*_2,32_ = 138.03, *P* < 0.001 for Mg; 2-way ANOVA with Tukey HSD post hoc test, *F*_2,32_ = 135.54, *P* < 0.001 for S). There was less variation in Mg and S foliar concentrations for *M. viridiflora* with only the highest treatment concentrations resulting in a significant increase in Mg and S concentration in both trials 1 and 2 (Fig. 2 and Table 2). Interestingly, the highest foliar Mg values for *M. viridiflora* were similar to the highest values in *A. excelsa*, at approximately 0.76%, yet *M. viridiflora* plants demonstrated a very different response in growth performance and health. The foliar concentration of S found in *M. viridiflora* receiving the highest MgSO_4_ treatment (15,300 mg l^−1^) was half that found in *A. excelsa* in the 9,100 mg l^−1^ treatment (approximately 0.6% c.f. 1.2%; Fig. 2b).

**Table 2:** *F*-statistics (with df. values in brackets) obtained from 2-way ANOVAs for foliar concentrations of Mg and S in *Melaleuca viridiflora* and *Alphitonia excelsa* exposed to different concentrations of MgSO_4_ over a 10 week period.

Element	Trial	Treatment	Leaf position	Treatment × leaf position
Mg (%)	1MV	(2,26) 32.86^***^	(1,26) 32.29^***^	(2,26) 9.466^***^
	2MV	(2,30) 5.91^**^	(1,30) 36.33^***^	(2,30) 3.219 ^ns^
	3AE	(2,32) 138. 03^***^	(1,32) 4.94^*^	(2,32) 0.98 ^ns^
S (%)	1MV	(2,26) 33.29^***^	(1,26) 17.65^***^	(2,26) 6.65^**^
	2MV	(2,30) 8.95^***^	(1,30) 10.29^**^	(2,30) 5.76^**^
	3AE	(2,32) 135.54^***^	(1,32) 4.35^*^	(2,32) 0.32 ^ns^

Key: 1MV = trial 1 *Melaleuca viridiflora*, 2MV = trial 2 *M. viridiflora*, 3AE = trial 3 *Alphitonia excelsa. P* values; ^ns^*P* > 0.05, ^*^P ≤ 0.05, ^**^P ≤ 0.01, ^***^P ≤ 0.001.

Overall, *M. viridiflora* had higher concentrations of Mg and S in lower leaves compared to upper leaves (e.g. In trial 1, 2-way ANOVA with Tukey HSD post hoc test, *F*_1,26_ = 32.29, *P* < 0.001 for Mg; 2-way ANOVA with Tukey HSD post hoc test, *F*_1,26_ = 17.65, *P* < 0.001 for S). In the 960 mg MgSO_4_ l^−1^ treatment *M. viridiflora* lower leaves had a Mg concentration of 0.76% compared with 0.52% in upper leaves. For *A. excelsa* upper leaves showed slightly elevated concentrations of Mg and S compared with lower leaves (Fig. 2, Table 2).

**Figure 3:**
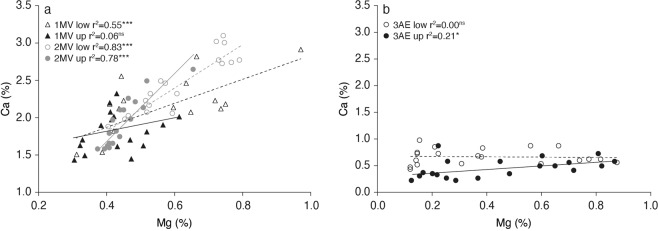
Relationship between Ca and Mg for leaves harvested from the upper (unfilled symbols) and lower (filled symbols) portion of plants from (**a**) trial 1 *Melaleuca viridiflora* (triangles), trial 2 *M. viridiflora* (circles), and (**b**) trial 3 *Alphitonia excelsa* (circles). *P* values; ^ns^*P* > 0.05, ^*^P ≤ 0.05^, **^P ≤ 0.01, ^***^P ≤ 0.001.

There was a significant positive relationship between foliar Mg and Ca concentrations in *M. viridiflora* (except for upper leaves in trial 1; Fig. 3a), and this relationship was strongest in trial 2. There was a weak positive relationship between Ca and Mg in the upper leaves of *A. excelsa*, however there was no relationship for the lower leaves (Fig. 3b).

## Discussion

Elevated concentrations of Mg and MgSO_4_ are emerging issues in land and water management^42^, with data urgently required to support informed management of contaminated water from RUM lease which occurs within Kakadu National Park. Our trials on *M. viridiflora* indicated that extremely high MgSO_4_ concentrations (~15,300 mg l^−1^) did not significantly affect leaf-scale physiological processes (stomatal conductance, chlorophyll fluorescence and predawn water potential), nor plant biomass of *M. viridiflora*. In contrast, we show that *A. excelsa* is a more susceptible species, with plant water status and plant biomass reduced by elevated concentrations of MgSO_4_ (~960 mg l^−1^), a significant outcome given the paucity of data previously available. Management of MgSO_4_ from mine waste rock and capping will need to consider species-specific responses to elevated MgSO_4_, with further research required on more species across a similar range of treatment concentrations.

A number of interrelated physiological mechanisms are likely to confer tolerance of elevated MgSO_4_ concentrations, as observed in *M. viridiflora*. The tolerance of a low Ca:Mg environment, and the relationship between these two ions is key to the response of plant species to elevated Mg concentrations^36^. Magela Creek water is very low in nutrients, with particularly low Ca concentrations of approximately 0.2–0.4 mg l^−1^ ^43^. As such, low Ca levels representative of those in Magela Creek were maintained as a constant across all treatments in the current study. With a low Ca:Mg ratio, Ca uptake can be competitively inhibited by Mg^44,45^, resulting in growth limitations due to the key role calcium has in plant cell formation^46^. However, this response is species dependent, with some species better adapted to low Ca:Mg environments^36^. The positive relationship between foliar concentrations of Ca and Mg for *M. viridiflora* indicates that it is tolerant of a low Ca:Mg ratio, adjusting Ca levels in response to the application of elevated MgSO_4_ concentrations.

The differing responses to elevated MgSO_4_ by the two study species may result from differing capacities to osmoregulate in response to this salt. The rapid negative response to high MgSO_4_ by *A. excelsa* suggests plants experienced osmotic stress^47^. In addition, the strong relationship between applied MgSO_4_ concentration and foliar concentrations of Mg and S (indicative of SO_4_ in leaves) in *A. excelsa* suggests this species does not exclude Mg or SO_4_ ions^48^. In contrast, in trial 1 *M. viridiflora* demonstrated little foliar accumulation of Mg or S with increasing MgSO_4_. This suggests root exclusion of ions may have occurred, as is commonly observed in salt tolerant plants^47,48^, at least for the lower range of concentrations. At higher concentrations (>9,100 mg MgSO_4_ l^−1^), it was evident that *M. viridiflora* was unable to fully exclude excess ions, as indicated by increasing foliar concentrations of Mg and S (Fig. 2a,b). However, this was limited to lower leaves, indicating translocation of ions to older leaves in order to maintain growth and function^49^. Thus, *M. viridiflora* exhibits mechanisms of root exclusion and translocation of excess ions, resulting in minimal negative response to elevated concentrations of MgSO_4_.

Root exclusion and translocation of ions, as inferred for *M. viridiflora*, are well described mechanisms for halophytic plants to manage salt balance^50^. There is evidence that *M. viridiflora* is tolerant of brackish water, with the species distribution within the Magela Creek catchment including reaches immediately upstream from mangrove stands (P. Christophersen, *pers. comms*.). Other common *Melaleuca* species, namely *M. cajuputi* and *M. leucadendra* may also have a similar tolerance to MgSO_4_ given the salt tolerance of *M. viridiflora*^51^. Such tolerant species would be suitable for riparian rehabilitation if dieback was observed due to elevated concentrations of MgSO_4_ in contaminated mine water from RUM. In contrast, *A. excelsa* does not extend into estuarine environments^52^ and its distribution is more representative of common tree species in the area, with the majority constrained to fresh water environments. Thus, testing additional species across a treatment regime informed by potential contamination concentrations is required for a comprehensive assessment of post-rehabilitation MgSO_4_ risks.

Our study showed that two common riparian trees from northern Australia have different tolerances to elevated concentrations of MgSO_4_, a mine water contaminant. It is likely that these differences are related to the relative salt tolerance of the two species, with the distribution of *M. viridiflora* indicating greater salt tolerance than *A. excelsa*. We infer that *M. viridflora* excludes uptake of Mg and SO_4_, and redistributes ions to older leaves. In contrast, *A. excelsa* demonstrated a lower tolerance to MgSO_4_, and is more likely to be impacted by increased MgSO_4_ levels in the environment. The outcomes of this work provide important information that will assist with mine site rehabilitation in an area surrounded by a World Heritage-listed national park, as well contribute to our understanding of plant response to elevated MgSO_4_ more broadly.

## Methods

### Study species

A glasshouse-based pot trial was undertaken at the University of Western Australia to determine the effect of elevated MgSO_4_ on two riparian tree species; *Melaleuca viridiflora* Sol. Ex Gaertn. and *Alphitonia excelsa* (Fenzl) Benth. Both species are widespread in the monsoonal wet-dry tropics of northern Australia, and occur in the riparian zone at Magela creek downstream of RUM in the Northern Territory (12.66°S, 132.89°E). *M. viridiflora* grows to 16 m and occurs in riparian habitats and seasonally inundated wetlands, and across a range of different soil types^53^. *A. excelsa* grows to 10 m and occurs across a broader range of habitats including riparian corridors, monsoon vine forests associated with permanent freshwater streams and savanna woodlands^52^. Temperatures at RUM range between 18 and 38 °C and the long-term average rainfall is 1,565 mm per year (Jabiru Airport 014198, Bureau of Meteorology, 2019). It is likely that riparian tree species are reliant on shallow groundwater (1 to 3.5 m below ground) during the dry season^54,55^.

### Experimental design

Three pot trials were undertaken (Table 3); trial 1 and 2 focussed on *M. viridiflora* and trial 3 focussed on *A. excelsa*. Each trial ran for 10 weeks, a period deemed long enough to detect the usually rapid response of plants to salinity and toxicity^56–58^. Treatments were applied daily as a liquid solution to each pot for 10 weeks. The liquid solution included a diluted Hoagland’s nutrient mixture (Supplementary Table 1) and each plant received 300 ml of solution per day. There is evidence that Ca ameliorates the effect of Mg on biota^36^. Previous ecotoxicology studies of aquatic biota in Magela Creek identified that a Ca:Mg of 1:9 has an ameliorating effect on the toxicity of Mg for biota from this location^43^. In this current study we maintained Ca concentration at 1 mg l^−1^, the background level at Magela Creek^43^, exceeding the 1:9 ratio for the majority of the treatments. This represents a worst case scenario where high levels of MgSO_4_ are released into the low Ca environment.

**Table 3:** Treatments applied in each trial showing both MgSO_4_ and Mg concentration, calculated osmotic water potential (Ψosm) and observed electrical conductivity (EC) of treatment solutions (*n* = 1).

	Species	MgSO_4_ (mg l^−1^)	Mg (mg l^−1^)	Ψosm (-kPa)	EC (mS/cm)
Trial 1	*Melaleuca viridiflora*	5	1	0.12	0.05
		15	3	0.35	0.07
		470	90	11.04	0.74
		960	190	22.55	1
Trial 2	*Melaleuca viridiflora*	6,000	12,00	140.93	6
		9,100	1,850	213.74	8
		15,300	3,100	359.37	11
Trial 3	*Alphitonia excelsa*	5	1	0.12	0.05
		960	190	22.55	1
		3,900	790	91.60	4
		9,100	1,850	213.74	8

*M. viridiflora* seedlings were sourced from a commercial nursery and *A. excelsa* plants were grown from seed in a glasshouse. Seedlings were removed from pots and all soil carefully washed from the roots. Seedlings of each species were transplanted into experimental pots of 9 cm diameter and 100 cm tall, filled with washed and steam-sterilised river sand, then acclimated for a minimum of two months in glasshouse conditions (30 °C/25 °C of diurnal/nocturnal temperature). Light level incident at the benchtop was ~1,990 µmol m^−2^ s^−1^ PAR at solar noon. Pots were positioned randomly within the glasshouse.

The range of MgSO_4_ treatment concentrations was chosen from baseline values in Magela Creek (approximately 1 mg l^−1^ for Mg and 0.78 mg l^−1^ for SO_4_), and historical observations of elevated concentrations from groundwater bores near the tailings facility (up to 417 mg Mg l^−1^ and 1,770 mg SO_4_ l^−1^)^25^. Trial 1 commenced when *M. viridiflora* plants were 10 months old with four treatments 5, 15, 470 and 960 mg l^−1^ MgSO_4_ (n = 6) (Table 3). Following trial 1, trial 2 commenced when plants were 12 months old and assessed the effect of three substantially higher concentrations 6,000, 9,100 and 15,300 MgSO_4_ (n = 6) due to the lack of detectable impact on *M. viridiflora* plants during trial 1. Trial 3 commenced when plants were 12 months old and tested the effect of 5, 960, 3,900 and 9,100 mg l^−1^ MgSO_4_ (Table 3) on *A. excelsa* (n = 5). The electrical conductivity (EC) of the applied solutions was measured using an Aqua-CP/A with Conductivity Sensor and a Vernier LabQuest 2 with Salinity Sensor for higher treatment concentrations (e.g. trial 2). The osmotic potential of treatment solutions was calculated based on the concentration of MgSO_4_ following Colmer *et al*.^49^.

### Leaf physiology

Plant vigour was assessed by measurements of leaf chlorophyll content, total plant dry weight, root:shoot ratio, concentration of key elements in leaf tissue (all trials), and measurements of stomatal conductance (g_s_), leaf chlorophyll fluorescence (Fv/Fm) and predawn plant water potential (ψ_pd_) (trials 2 and 3). All measurements were made at the end of the trial in week 10.

Leaf chlorophyll content was assessed using a colorimeter (SPAD502Plus, Konica Minolta Pty, (SPAD)). We quantified the chlorophyll content in leaves across the full range of measured SPAD values (n = 18 and 20 for *A. excelsa* and *M. viridiflora* respectively) following the methods of Hendry and Grime^59^ and the relationship between SPAD values and chlorophyll content (r^2^ = 0.74, *P* < 0.001 and r^2^ = 0.63 and *P* < 0.001 for *M. viridiflora* and *A. excelsa* respectively) was used to determine leaf chlorophyll content (Supplementary Fig. 2). Fv/Fm was measured on dark-adapted leaves using a Pocket PEA (Hansatech Instruments) and g_s_ was measured with a leaf porometer (SC-1 Decagon). Fv/Fm and g_s_ were measured on four leaves from each replicate plant between 08:30 and 11:30AM local time. Predawn leaf water potential was measured using a Scholander-type pressure chamber (Model 600, PMS Instrument Company) on a small twig for *M. viridiflora* and one leaf for *A. excelsa*, sampled from the upper (younger) portion of each replicate plant. For predawn water potential, Fv/Fm, g_s_ and chlorophyll content there were 6 replicate plants for trial 2 and 5 replicates for trial 3, except at the higher treatment levels (3,900 and 9,100 MgSO_4_ mg l^−1^) because most leaves had abscised or desiccated, therefore measurements were limited to a subset of replicates (n = 4 and 1 respectively). The treatment with only one replicate (3,900 MgSO_4_ mg l^−1^) was not included in the analysis.

Nutrient content was determined for upper and lower leaves in week 10 for *M. viridiflora*, and week 7 for *A. excelsa* when it was evident that leaves were abscising from the higher treatment plants. Dried samples were ground, acid digested and the concentrations of major ions were analysed using ICP-OES. MgSO_4_ in solution dissociates into Mg and SO_4_, thus foliar S concentrations are considered indicative of SO_4_ concentration, with SO_4_ the only applied source of S. All plants were destructively sampled at the end of the trials, and sand was carefully washed from the root material. Leaf, stem and root material was dried at 60 °C until mass stabilised and dry mass of each component was determined.

For leaf physiological variables (Fv/Fm, stomatal conductance, predawn water potential and chlorophyll content) differences between treatments within each trial was tested using one-way analysis of variance (ANOVA) with Tukey honestly significant difference (HSD) post hoc test. For foliar concentrations of Mg and S, 2-way ANOVAs were used to test for differences between MgSO_4_ treatments and between upper and lower leaves. Homogeneity of variance was tested using Levene’s test and normality of data distribution was determined through Shapiro-Wilk test and a visual assessment of the residuals. ANOVAs were on untransformed data, except for water potential for *A. excelsa* and foliar Mg content for *M. viridiflora* in trial 2, with analyses instead performed on log-transformed data. The relationships between foliar concentrations of Ca and Mg were determined using linear models. All analyses were completed in R 3.5.2^60^.

## Supplementary information

Supplementary Information.

## Data Availability

Data is available through the University of Western Australia’s research repository (https://research-repository.uwa.edu.au/en/datasets/).
