# Supplementary material for: Effect of elevated magnesium sulfate on two riparian tree species potentially impacted by mine site contamination
Source: Sci Rep. 2020 Feb 19;10:2880. doi: 10.1038/s41598-020-59390-9 (PMC7031394; doi:10.1038/s41598-020-59390-9)

Tolerance of elevated magnesium sulphate of two riparian tree species impacted by mine site contamination

Caroline A. Canham, Ornela Y. Cavalieri, Samantha A. Setterfield, Fiona L. Freestone, Lindsay B. Hutley

Supplementary Table 1. Summary of nutrient solution composition.

Concentrations of nutrients in solution that was combined with $MgSO_4$ treatment concentrations, with 300 ml of solution delivered to each plant daily.

Supplementary Figure 1. Representative plants after 10 weeks of treatment.

Trial 1 - *Melaleuca viridiflora*; a) 5 mg l^{-1} $MgSO_4$; b) 960 mg l^{-1} $MgSO_4$; trial 2 - *M. viridiflora*; c) 6,000 mg l^{-1} $MgSO_4$; d) 9100 mg l^{-1} $MgSO_4$; trial 3 - *Alphitonia excelsa*; e) 5 mg l^{-1} $MgSO_4$; f) 960 mg l^{-1} $MgSO_4$; g) 3900 mg l^{-1} $MgSO_4$; h) 9100 mg l^{-1} $MgSO_4$.

Supplementary Figure 2. Relationship between SPAD values and chlorophyll content.

Chlorophyll content in leaves was quantified across the full range of measured SPAD values (n = 18 and 20 for *Alphitonia excelsa* and *Melaleuca viridiflora* respectively). The relationship between SPAD values and chlorophyll content (y = 0.0002x–0.0024 for *M. viridiflora* and y = 0.0003x-0.002 for *A. excelsa*) was used to determine leaf chlorophyll content. Key: MV = *Melaleuca viridiflora*; AE = *Alphitonia excelsa*, *** = $P \leq 0.001$.

Supplementary Table 1.

**Compound**	**Nutrient**	**mg l^{-1}**
$CaCl_2.2H_2O$	Cl	1.773
	Ca	1.002
$KNO_3.0H_2O$	K	7.820
	NO_3	12.400
$KH_2PO_4.0H_2O$	K	0.020
	PH_4	0.470
$MnSO_4.H_2O$	Mn	0.007
	SO_4	0.012
$ZnSO_4.7H_2O$	Zn	0.003
	SO_4	0.005
$CuSO_4.5H_2O$	Cu	0.0006
	SO_4	0.0009
H_3BO_3	BO_3	0.071
$Na_2MoO_4.2H_2O$	Mo	0.001
	Na	0.0007
FeNaEDTA.0H2O	Fe	0.056
	Na	0.023
$MgSO_4.7H_2O$	Mg	According to treatment
	SO_4	According to treatment

Supplementary Figure 1.

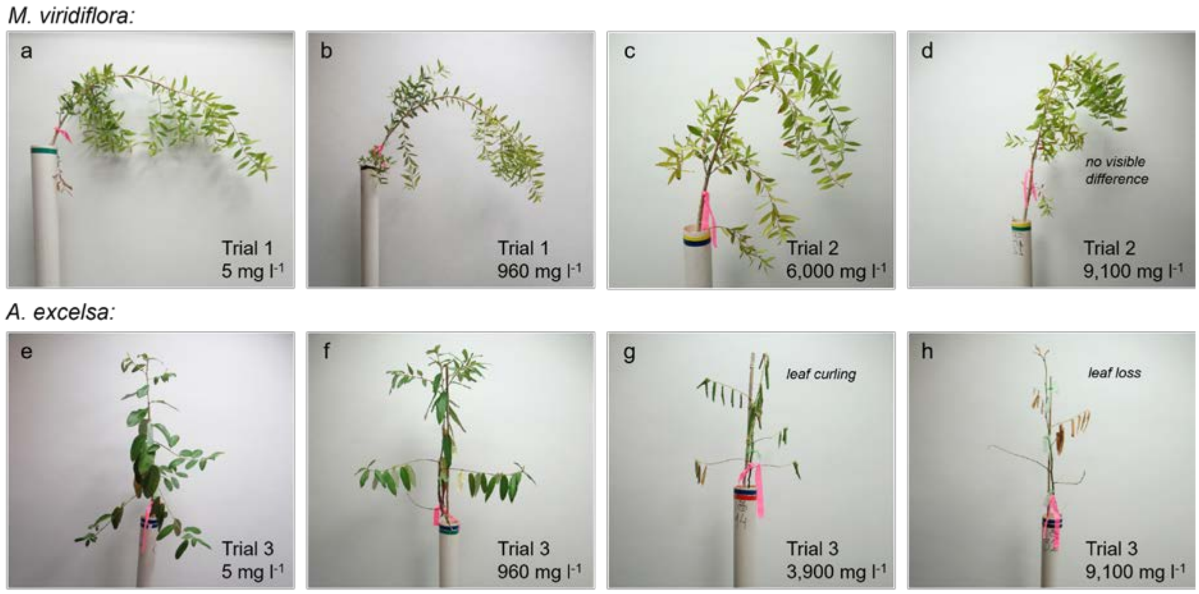

Supplementary Figure 2.

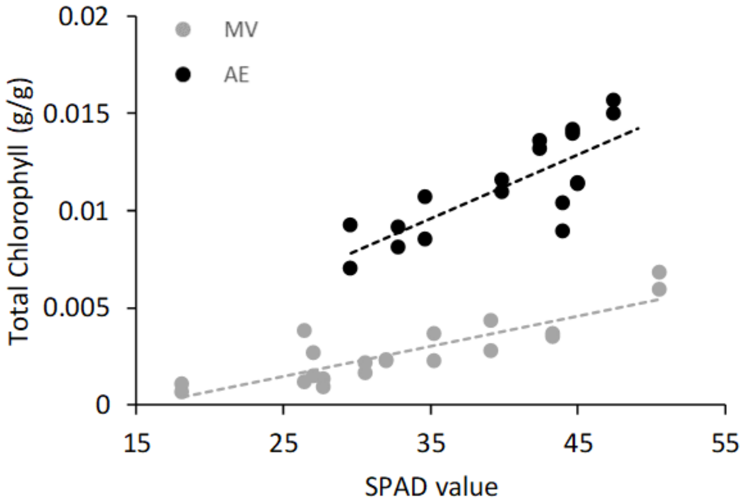

Supplement: Supplementary file 1 — Supplementary Information. [file 41598_2020_59390_MOESM1_ESM.pdf]
